# Virtual reality experiential learning improved undergraduate students’ knowledge and evaluation skills relating to assistive technology for older adults and individuals with disabilities

**DOI:** 10.1186/s12909-024-05085-y

**Published:** 2024-01-30

**Authors:** Peng-Hsu Chen, Hsuan-Wei Ho, Hung-Chou Chen, Ka-Wai Tam, Ju-Chi Liu, Li-Fong Lin

**Affiliations:** 1 School of Medicine, College of Medicine, Taipei Medical University, Taipei, 110 Taiwan; 2 Department of Physical Medicine and Rehabilitation, School of Medicine, College of Medicine, Taipei Medical University, Taipei, 110 Taiwan; 3 Department of Physical Medicine and Rehabilitation, Shuang Ho Hospital, Taipei Medical University, New Taipei City, 235 Taiwan; 4 Shared Decision Making Resource Center, Shuang Ho Hospital, Taipei Medical University, New Taipei City, 235 Taiwan; 5 Cochrane Taiwan, Taipei Medical University, Taipei, 110 Taiwan; 6 Division of General Surgery, Department of Surgery, Shuang Ho Hospital, Taipei Medical University, New Taipei City, 235 Taiwan; 7 Division of General Surgery, Department of Surgery, School of Medicine, College of Medicine, Taipei Medical University, Taipei, 110 Taiwan; 8 Division of Cardiology, Department of Internal Medicine, Shuang Ho Hospital, Taipei Medical University, New Taipei City, 235 Taiwan; 9 Taipei Heart Institute, Taipei Medical University, Taipei, 110 Taiwan; 10 Division of Cardiology, Department of Internal Medicine, School of Medicine, College of Medicine, Taipei Medical University, Taipei, 110 Taiwan; 11 School of Gerontology and Long-Term Care, College of Nursing, Taipei Medical University, 250 Wu-Hsing Street, Taipei, 110 Taiwan; 12 Research Center for Artificial Intelligence in Medicine, Taipei Medical University, Taipei, 110 Taiwan; 13 Neuroscience Research Center, Taipei Medical University, Taipei, 110 Taiwan

**Keywords:** Virtual reality, Assistive technology, Experiential learning, Undergraduate student, Gerontology, Long-term care

## Abstract

**Background:**

The aging population has caused assistive technology (AT) to receive attention. Thus, ensuring accurate user comprehension of AT has become increasingly crucial, and more specialized education for students in relevant fields is necessary. The goal of this study was to explore the learning outcomes in the context of AT for older adults and individuals with disabilities through the use of VR experiential learning.

**Methods:**

A parallel-group design was used. Sixty third-year university students studying gerontology and long-term-care-related subjects in Taiwan were enrolled, with the experimental (VR) and control (two-dimensional [2D] video) groups each comprising 30 participants. Both groups received the same 15-minute lecture. Subsequently, the experimental group received experiential learning through a VR intervention, whereas the control group watched a 2D video to learn. The students’ knowledge of AT was assessed using a pretest and posttest. Additionally, their skills in evaluation of residential environments were assessed using the Residential Environment Assessment (REA) Form for Older Adults. All data analyses were performed with SPSS version 22.

**Results:**

In the posttest conducted after the intervention, the experimental group exhibited a significant 20.67 point improvement (*p* < 0.05), whereas the control group only exhibited improvement of 3.67 points (*p* = 0.317). Furthermore, the experimental group demonstrated a significantly higher score (+ 2.17 points) on the REA Form for Older Adults than did the control group (*p* < 0.05).

**Conclusion:**

VR experiential learning can significantly improve undergraduate students’ knowledge and evaluation skills in relation to AT for older adults and individuals with disabilities.

## Introduction

Experiential learning (or hands-on learning) is the concept of “learning by doing” suggested by American educator John Dewey in the early 20th century. In the 1980s, American educational theorist David Kolb published the Kolb Experiential Learning Cycle Theory, which was based on Dewey’s theory. Kolb proposed that learning is a process of acquiring knowledge through the transformation of experience, which involves four cyclic stages starting with concrete experience (CE), moving to reflective observation (RO), abstract conceptualization (AC), and finally active experimentation (AE). In this cycle, the CE and AC stages constitute the process of experience, and RO and AE constitute transformation [1]. The high effectiveness of virtual reality (VR) learning is strongly related to the effective application of experiential learning theory [2, 3]. VR is an emerging technology and can be immersive or nonimmersive. Immersive virtual reality (IVR) usually generates a simulated environment by using a VR viewer with a head-mounted display (HMD) [4], creating an immersive and interactive experience for users [5]. It also provides a safe, controllable, and repeatable environment for the performance of real-world activities, enabling users to experience and interact with the complex real world within a virtual one [6].

VR has been widely used in teaching [7]. It enables young students to be more active [8–10] and experience deeper feelings than usual during interactive learning [11, 12]. Through this type of learning, learners more effectively and fully memorize material [13–15]. Furthermore, VR technology has been widely used in many fields of medical education, including medical student education, nursing, anatomy, surgery, infection control, and radiology. In the teaching of medical students, VR use can improve learning quality, knowledge, and motivation [16–18]; in nursing education, VR use can improve knowledge [19, 20]; in anatomy education, VR use can significantly improve medical students’ performance in anatomy courses [21, 22]. In addition, VR use can assist the teaching of traditional anatomy courses [23, 24] and is used in many aspects of clinical medicine [25]. Surgical processes can be taught completely using VR [26–29], which can also improve surgical technique and patient outcomes [30]. Regarding infection control education, VR use can improve students’ academic performance and learning motivation [31], whereas regarding radiology education, VR technology enables students to effectively acquire skills and learn [32] and to participate more actively [33].

The international definition of assistive technology (AT) is as follows: “Tools, techniques, or environments, which can maintain or improve the limitation of daily activities caused by functional impairment” [34]. The benefits of using ATs, especially those used for mobility, are that they improve the user’s activities of daily living, independence, quality of life, social well-being, confidence, and self-esteem [35–40]. With the aging population and the increasing recognition that the use of AT can promote healthy aging, ensuring that users understand AT has become essential [41–43]. The assistance of a certified AT professional can improve an individual’s outcomes in their use of AT [44], including the selection of the correct AT [45]. However, some AT professionals believe that their training does not meet the required standards [46]. Furthermore, previous research has also highlighted that the effectiveness of learning AT through traditional methods was insufficient [47]. Therefore, education for AT professionals should be increased, including for undergraduates, and various teaching methods must be studied.

To the best of our knowledge, this study was standing as the first attempt to use VR as a teaching tool in the field of AT for older adults. Therefore, this study was conducted on the basis of experiential learning theory. HMD-IVR technology and traditional AT courses, which address assistive devices and barrier-free environments for older adults and individuals with disabilities, were utilized; this enabled us to compare the learning effects, including on knowledge and evaluation skills, of different teaching methods. The goal of this study was to explore the learning outcomes in the context of AT for older adults and individuals with disabilities through the use of VR experiential learning.

## Methods

### Participants

Sixty third-year university students studying gerontology and long-term-care-related subjects in Taiwan, including 30 students from the 2020–2021 academic year and 30 from the 2021–2022 academic year, were enrolled. They agreed to participate in the experiment and had not yet received clinical training. Students were excluded if they could not complete the questionnaire administered or had a physical disability or injury that prevented them from participating in simulation training. They were divided between two groups by academic year: the experimental (VR) group comprised the 30 students in the 2021–2022 academic year, and the control (video) group comprised the 30 students in the 2020–2021 academic year.

### Protocol

**Fig. 1:**
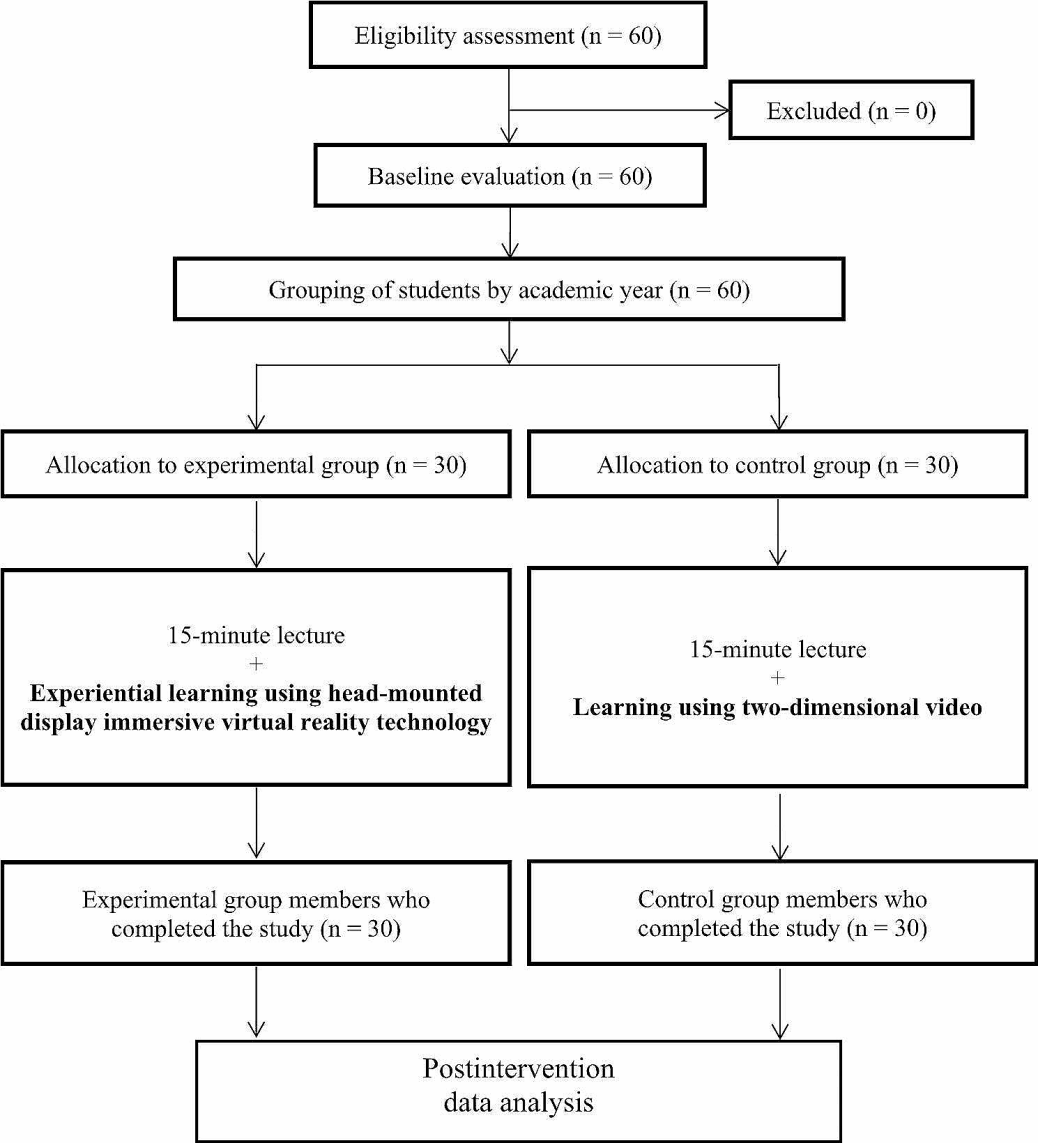
Study flowchart

An online pretest was administered before the course that consisted of two parts (basic information and a test of professional knowledge). On the day of the course, the speaker gave the students a 15-minute lecture. The lecture introduced the design principles of AT and the practices of Taiwan’s Long-Term Care Plan 2.0. Subsequently, the experimental group received experiential learning through a VR intervention, where students could take turns using VR to engage with the course content, while the teacher provided guidance on how to operate head-mounted display–immersive virtual reality (HMD-IVR) technology. In contrast, the control group collectively watched a two-dimensional (2D) video for their classroom learning, while the teacher conducted group instruction in the classroom. The VR course is an original curriculum, created through collaboration between the teacher offering expertise and virtual scene designers with modeling capabilities provided by Taipei Medical University. The content of the 2D video was the same as that of the VR material. However, the students in the control group did not experience immersive and interactive learning and could not pause the video. After the interventions, the groups took a posttest on their professional knowledge and completed a Residential Environment Assessment (REA) for Older Adults Form for skills evaluation. In addition, the experimental group responded to open-ended questions that were in the predesigned VR Experience Worksheet (Fig. 1).

### VR intervention

**Fig. 2:**
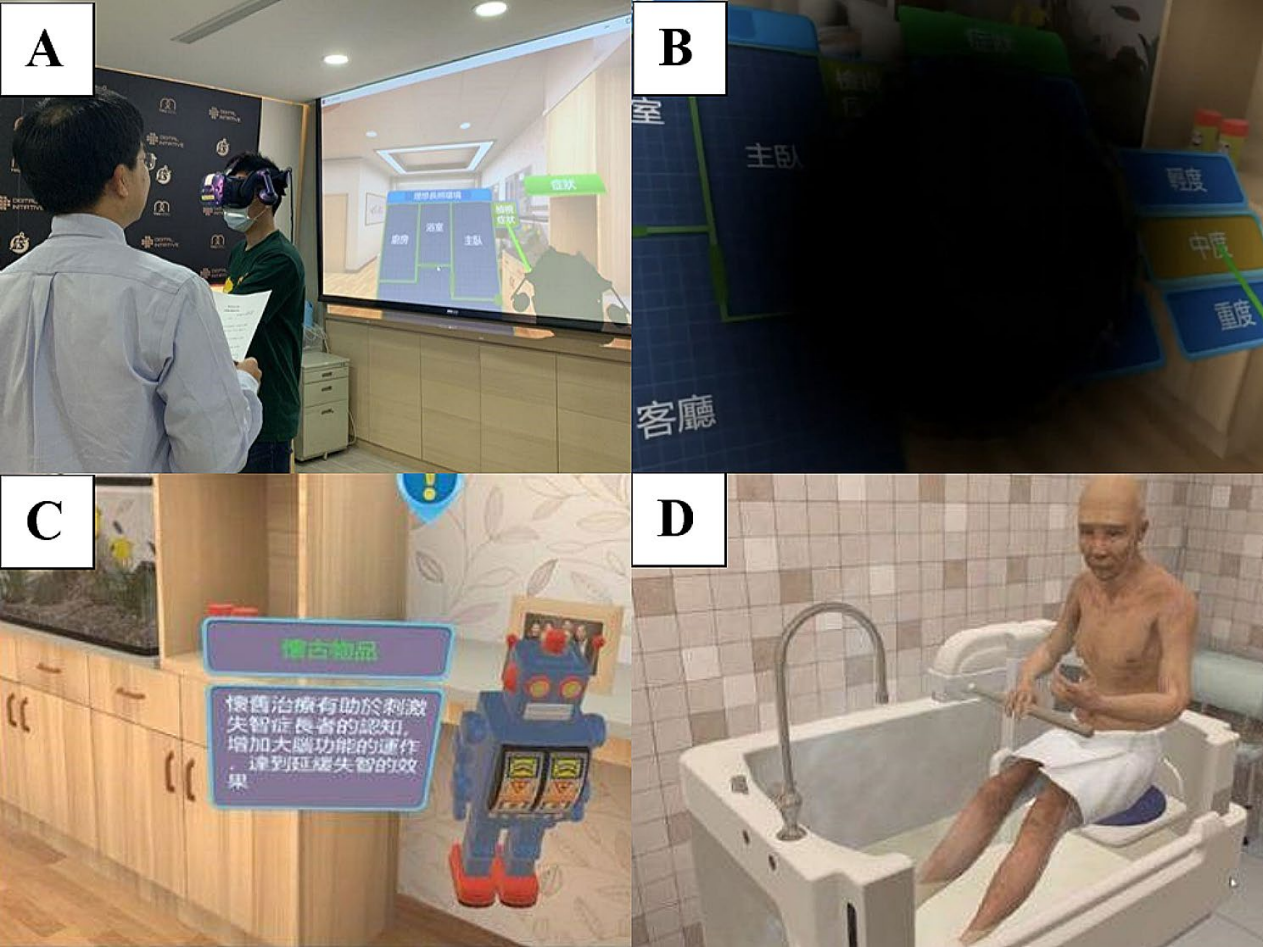
Setup of virtual reality intervention: (**A**) using head-mounted display–immersive virtual reality for student learning; (**B**) simulation of moderate macular degeneration; (**C**) text-based explanation interface providing detailed explanation of the design concept and usage; and (**D**) application of assistive technology to a virtual character in a bathroom

The experimental group used HMD-IVR technology to undertake VR learning that was based on experiential learning theory (Fig. 2a). The VR learning was complemented using the VR Experience Worksheet and involved a simulation of visual impairment, experiencing mobile indoor space patterns, watching a video on the design and use of AT, and searching for furniture and equipment. The Virtual Reality Experience Worksheet comprised open-ended questions that evaluated the student’s experience of VR learning.

The scene design of the VR course had three major features (Table 1). The first enabled the students to experience an aging-related decline in sight through VR. This was achieved using a special-effect lens filter, which covered the students’ field of vision. The filter produced the effect of visual impairment by distorting the line of sight, increasing the number of visual barrier points, and reducing the field of vision, enabling simulation of conditions such as cataracts, macular degeneration and glaucoma and enabling students to experience the inconvenience of the visual impairment common in older adults (Fig. 2b).

**Table 1:** The three main features of the scene design in the VR course

Feature of VR experiential learning	Implementation	Simulated Conditions	Advantage
Visual impairment experience	Special-effect lens filter	1. Cataracts 2. Macular degeneration 3. Glaucoma	Students experience the inconvenience of visual impairment common in older adults
Barrier-free living environment and assistive devices	VR environment with four main living spaces and 20 items* of AT	1. Walk around freely to observe the design of the space 2. Text-based explanation interface of items of AT 3. Virtual character using items of AT	Students experience age-friendly living spaces and learn about assistive devices from first-person perspective
Interactive experience	Physiological responses of the virtual character	1. Blinked and looked at the students regularly 2. Neck and head moved slightly to face the students 3. Mouth moved when the character was speaking	Students engage in natural interactions, providing a real-world experience

VR: virtual reality; AT: assistive technology

*: The detailed description of 20 items of AT is provided in Table 2

The second feature enabled simulation of a barrier-free living environment and assistive devices; VR enabled the presentation of an ideal living environment for older adults, including four main living spaces (living room, kitchen, bathroom, and bedroom) and 20 items of AT (Table 2) designed for older adults. During the learning process, the students could walk around freely to observe the design of the space, and a text-based explanation interface provided a detailed explanation of the concepts and uses of the 20 items of AT (Fig. 2c). For some key objects, students could also watch a virtual character using the object from different points of view within VR (Fig. 2d), which more directly conveyed the concept of an age-friendly living environment than did observation solely from the first-person perspective. A free viewing mode was adopted in the VR course; the students could freely move from the space they were located in after entering the virtual world, read the descriptions of the objects that they wished to learn about, and experience an age-friendly living environment. Furthermore, in the wheelchair experience that was included, each VR room was combined in a seamless open space to provide students with a larger activity space. Therefore, the students could use a wheelchair to move between the living room, kitchen, bedroom, and other spaces to experience life using a wheelchair.

**Table 2:** Four main living spaces and items of assistive technology (AT) designed for older adults

Main living spaces	Items of assistive technology (AT)
Living room	1. Antique items 2. Rise-assist cushion 3. Emergency call bell 4. Bi-directional opening door
Kitchen	1. Rounded dining table 2. Adapted tableware 3. Designed reflective mirror 4. Underneath the tabletop designed with a hollow space (kitchen sink counter, touch-controlled double induction cooker) 5. Extendable faucet 6. Foot-operated water pedal 7. Emergency call bell
Bathroom	1. Extendable faucet 2. Bathroom sliding door 3. Accessible drainage hole 4. Assistive toilet seat cushion 5. Tilted vanity mirror 6. Bathtub lift 7. Bathroom chair with attached suction cups 8. Emergency call bell
Bedroom	1. Electrically adjustable cabinet 2. Emergency call bell 3. Modified wardrobe 4. Electrically adjustable bed 5. Modified clothing rack 6. Bi-directional opening door

The third feature enabled a more interactive experience; simple physiological responses were designed and added to the virtual character so that the virtual character closely resembled a real person. This design enabled the students to interact more naturally with the AT in the virtual world and thus provided a more immersive training experience. The physiological responses were mainly divided into the following: the virtual character blinked and looked at the students regularly; the neck and head moved slightly to face the students; and the mouth moved when the character was speaking (the mouth shape did not correspond to the content of the speech). In addition, because the text-based explanation interface in the VR was movable, the students were required to continually confirm their understanding of the goals and actions of the current step and read the text-based explanations during the learning process. Therefore, it was necessary to design a convenient and clear explanation interface for reading content within the VR.

### Outcome assessment

The experiment measured two primary outcomes, professional knowledge and evaluation skills, and one secondary outcome, responses to open-ended questions regarding the VR experience.

### Primary outcome

#### Knowledge of AT

The professional knowledge of the students was assessed using a pretest and posttest. The content of the pretest and posttest was identical; they comprised 10 multiple-choice questions related to the living environment of older adults (10 points per question, 100 points in total). The questions were as follows:

1. Which of the following is not the most common environmental problem in a general household?
  A. Narrow living space
  B. Inconvenient bathroom and toilet facilities
  C. Slippery floors, uneven surfaces
  D. **Overexposure to sunlight**

2. The visual symptoms of this type of impairment are that objects appear blurry regardless of whether they are far or near, objects appear to have layers and afterimages, the contrast and vividness of sight are reduced, and everything appears darker. What type of visual impairment is this?
  A. **Cataract**
  B. Macular degeneration
  C. Glaucoma
  D. Diabetic retinopathy

3. What are the two major parts of the home environment assessment for older adults? Which of the following is correct?
  A. Prevention and accessibility
  B. Monitoring and control
  C. Safety and accessibility
  D. **Monitoring and Safety**

4. The visual symptoms of this type of impairment include blurred vision and central vision impairment. The more severe the symptoms, the greater is the range of the visual impairment. What type of visual impairment is this?
  A. Cataract
  B. **Macular degeneration**
  C. Glaucoma
  D. Diabetic retinopathy

5. To assist a person with poor physical function in lying down and sitting up, which part of the backboard of the electric bed must be raised first?
  A. Head
  B. Upper back
  C. **Knee**
  D. Lower back

6. The visual symptoms of this type of impairment are that the peripheral vision begins to blur and that the more severe the symptoms, the greater is the range of the visual impairment. What type of visual impairment is this?
  A. Cataract
  B. Macular degeneration
  C. **Glaucoma**
  D. Diabetic retinopathy

7. Regarding the function of the electric bed, which of the following is correct?
  A. The bed board angle can be controlled (adjusting the angles of the back and knees), making it convenient for sitting up and lying down.
  B. The height of the bed surface can be adjusted for the convenience of caregivers to provide care services.
  C. Equipped with movable dual-side guardrails to enhance safety during lying down, turning, sitting up, or standing up.
  D. **All of the above**

8. If an older adult has difficulty grasping eating utensils with their fingers, what form of handle should the eating utensils have to reduce the finger manipulation required?
  A. **Thicker**
  B. Slimmer
  C. Thinner
  D. Lighter

9. To enable older adults to cook as much as possible, what kitchen appliance—that most strongly related to the convenience of cooking—can be used?
  A. Countertop
  B. Mirror
  C. Electric lift cabinet
  D. **All of the above**

10. For those with insufficient hand dexterity, what form of chopsticks can be used to reduce the requirement of hand movement control and facilitate eating?
  A. Pinch method
  B. Rest method
  C. **Clamp method**
  D. Hold method

#### Evaluation skills for REA

Teachers measured the students’ evaluation skills in their use of the REA Form for Older Adults. The scoring system utilizes a Likert scale with a five-point rating, categorizing evaluation skills as very poor, poor, moderate, good, and very good. There are eight main questions, each subdivided based on specific sub-items. The scoring distribution for each main question varies, and the percentage allocation is indicated after each question. The total score reached 100 points. The form collected the following:

1. Basic case information. (5 points)
2. The body structure and function of the individual: medical diagnoses related to the use of assistive devices; vision; visual perception; the optimal weather and time for visibility; light and dark adaptation; hearing; gross motor skills; fine motor skills; and particular habits or hobbies. (10 points)
3. The daily activity and role of the individual: their role in their family; and whether the individual requires assistance in undertaking daily activities. (10 points)
4. The living situation of the individual: whether they are a main caregiver, their living situation, their apartment type, and their floor covering. (5 points)
5. The individual’s main at-home mobility or movement assistive devices when moving horizontally (including stepping over thresholds) and vertically: handrails, a single cane, two canes, crutches, underarm crutches, forearm crutches, a walker, a manual wheelchair, an electric-powered wheelchair, lifts, ladders, or others. (5 points)
6. Assessment of the home environment and assistive devices of the individual. (5 points)
7. Evaluation of the individual’s current situation: bedroom space (door panels, thresholds or height differences, slopes, color contrast, lightness or darkness, and slippery floors), residential gate and residential access (door panels, thresholds and height differences, color contrast, lightness or darkness, slippery floors in front of doors, handrail settings, aisle widths, stairs, and slopes), bathroom space (doorway, interior space, bathtub, toilet, and washbasin), kitchen space (door panels, threshold or height difference, slope, color contrast, lightness or darkness, height of countertop, space under the countertop, faucet, range hood, handrail setting, and slippery floor). (30 points)
8. Floor plan of the home environment, relevant descriptions of its use, and suggestions for improvement (it was recommended that the location of each space and individual flows of movement be indicated; If a space was identified for improvement, the size of the space and addition or change to be made could be indicated; furthermore, photos could be attached to aid explanation. If many rooms required improvement, the necessary changes for each room had to be listed individually). (30 points)

Those scoring the REA Form for Older Adults were two professionals in the field of assistive devices for older adults.

### Secondary outcome

The responses to the open-ended questions in the VR Experience Worksheet were assessed as the secondary outcome. The questions included the following:

1. How did you feel while wearing and using the virtual reality equipment?
2. What aspects of the virtual reality technology applied in this course were impressive (e.g., spatial objects, equipment, or the presentation of visual impairment)?
3. How are virtual reality technology and medical care associated?
4. In addition to the field of medical care, what possible areas could virtual reality be applied to in the future?

### Statistical analysis

The basic information of the participants—including age, sex, computer usage experience, and VR usage and learning experience—and the scores from the pretest, posttest, and REA Form for Older Adults were analyzed using descriptive statistics. The Shapiro–Wilk test was used to verify whether the variables were normally distributed. We compared the intergroup differences in sex, computer usage experience, and VR usage and learning experience by using the chi-square test and compared the score difference between the pretest and posttest using the paired *t* test. We compared the groups’ age, pretest scores, posttest scores, and REA Form for Older Adults scores by using the independent *t* test. All data analyses were performed with SPSS version 22, and α < 0.05 indicated significance.

## Results

### Participant demographics

A total of 60 third-year university students studying gerontology and long-term-care-related subjects met the inclusion criteria and provided consent to participate in this experiment. They were divided into two groups of 30 participants: the experimental (VR) group and the control (video) group. The demographic characteristics of the participants are listed in Table 3. In the experimental group, the participants’ ages ranged from 20 to 22 years, with an average of 20.17 ± 0.38 years. The proportion of men in the group was 33%. Of the experimental group, 6.7% had computer usage experience of less than 2 years, 3.3% had computer usage experience of 2 to 4 years, 16.7% had computer usage experience of 4 to 6 years, 30% had computer usage experience of 6 to 10 years, and 43% had computer usage experience of more than 10 years. Of the experimental group, 50% had VR usage experience and 50% had VR learning experience. In the control group, the participants’ ages ranged from 20 to 22 years, with an average of 20.30 ± 0.54 years. The proportion of men in the group was 50%. Of the control group, 0% of participants had computer usage experience of less than 2 years, 6.7% had computer usage experience of 2 to 4 years, 10% had computer usage experience of 4 to 6 years, 20% had computer usage experience of 6 to 10 years, and 63.3% had computer usage experience of more than 10 years. In the group, 43.3% had VR usage experience and 40% had VR learning experience. We analyzed the demographic characteristics of the participants in both groups, including their age, sex, computer usage experience, VR usage experience, and VR learning experience. The two groups did not differ significantly (*p* > 0.05; Table 3).

**Table 3:** Demographic characteristics

General characteristics	Experimental	Control	*p*
Participants (number)	30	30	
Age (mean ± SD, years)	20.17 (0.38)	20.30 (0.54)	0.270
Sex (n, %)			0.190
Men	10 (33.3%)	15 (50%)	
Women	20 (66.6%)	15 (50%)	
Computer usage experience (n, %)			0.336
< 2 years	2 (6.7%)	0 (0%)	
2–4 years	1 (3.3%)	2 (6.7%)	
4–6 years	5 (16.7%)	3 (10%)	
6–10 years	9 (30%)	6 (20%)	
> 10 years	13 (43.3%)	19 (63.3%)	
VR usage experience (n, %)	15 (50%)	13 (43.3%)	0.605
VR learning experience (n, %)	15 (50%)	12 (40%)	0.436

VR: virtual reality; Experimental: VR intervention group; Control: 2D video intervention group; SD: standard deviation

### Primary outcome

#### Knowledge of AT

The experimental group scored an average of 66.33 ± 17.71 points in the pretest and 87.00 ± 13.17 points in the posttest. The group exhibited a 20.67-point improvement, and this difference was significant (*p* < 0.05). The control group scored an average of 63.00 ± 10.88 points in the pretest and 66.67 ± 16.68 points in the posttest. The group exhibited a 3.67-point improvement, and this was not a significant difference (*p* = 0.317). The pretest scores of the experimental (66.33 ± 17.71) and control (63.00 ± 10.88) groups did not differ significantly (*p* = 0.384). However, the posttest scores of the experimental (87.00 ± 13.17) and control (66.67 ± 16.68) groups did differ significantly (*p* < 0.05; Table 4).

**Table 4:** Within-group and between-group comparisons of knowledge of AT in experimental (*n* = 30) and control (*n* = 30) groups at the pretest and posttest

		Experimental			Control			Between-group *p*
		Mean (SD)	Within-group change (SD)	Within-group *p*	Mean (SD)	Within-group change (SD)	Within-group *p*	
Knowledge of AT	Pretest	66.3 (17.7)	20.7 (16.8)	0.000*	63.0 (10.9)	3.7 (19.7)	0.317	0.384
	Posttest	87.0 (13.2)			66.7 (16.7)			0.000*

Values are expressed as mean ± standard deviation. *p < 0.05 using paired *t* test for within-group comparisons of pretest and posttest. *p < 0.05 using independent *t* test for between-group pretest and posttest comparisons. Experimental: VR intervention group; Control: 2D video intervention group; SD: standard deviation; AT: assistive technology

#### Evaluation skills for REA

The experimental group scored an average of 89.10 ± 3.04 points for the skills evaluation, and the control group scored an average of 86.93 ± 4.43 points. The experimental group exhibited a 2.17-point higher score than did the control group, and this was a significant difference (*p* < 0.05; Table 5).

**Table 5:** Differences in skills of evaluation of a residential environment between experimental (*n* = 30) and control (*n* = 30) groups

	Experimental mean (SD)	Control mean (SD)	Between-group p
REA Form for Older Adults score	89.1 (3.0)	87.0 (4.4)	0.032*

Values are expressed as mean ± standard deviation. *p < 0.05 using independent *t* test for between-group REA score comparison. Experimental mean: virtual reality intervention group mean score; Control mean: 2D video intervention group mean score; SD: standard deviation; REA: Residential Environment Assessment

### Secondary outcome

We have roughly consolidated open-ended responses of four questions of VR Experience Worksheet from 30 students and provided a few common answers as examples.

The answers to the first open-ended question on the VR Experience Worksheet, “How did you feel while wearing and using the virtual reality equipment?”, included the following:

1. *I became dizzy and nauseous while using the HMD-IVR, but after taking a short break, I could continue and complete the course.*
2. *The VR was very realistic, very interesting, and easy to operate.*
3. *HMD-IVR device was too heavy.*

The answers to the second question, “What aspects of the virtual reality technology applied in this course were impressive (e.g., spatial objects, equipment, or the presentation of visual impairment)?”, included the following:

1. *VR technology makes me feel as if I’m actually present in a particular environment and enables me to observe and operate assistive devices.*
2. *The visual impairment filter showed me how the world appears to older people with visual impairment; I had empathy because of the immersive technology, and I would not have gotten that through lectures.*
3. *By using the VR technology, I could more deeply understand the course’s content.*

The answers to the third question, “How are virtual reality technology and medical care associated?”, included the following:

1. *When I used the VR technology, I found that it was easier to remember the symptoms of a disease. It also helped me feel empathy for older people because I could experience the disabilities they faced.*
2. *Before I used the VR immersive technology, it was tough for me to tell the differences between the types of visual impairment in the pretest, but once I had used the VR, it was a lot easier to understand because I could see everything up close and personal. In the posttest, I did much better because of the VR technology.*

The answers to the final question, “In addition to the field of medical care, what possible areas could virtual reality be applied to in the future?”, included the following:

*Traveling, exercise, psychotherapy, driving license exams, building, and more.*

## Discussion

To our knowledge, this was the first attempt to use IVR experiential learning as an educational intervention in the field of AT. It was novel that VR experiential learning can improve undergraduate students’ knowledge and evaluation skills in relation to AT for older adults and individuals with disabilities. especially significant improvement in knowledge was noted.

In consideration of the impending super-aged society, this study is valuable because it enhanced students’ understanding of aging-related problems and helped prepare them for the increasing demand in care of older adults. The main teaching aim of this course was to provide effective training to students to bridge the gap between academic learning and clinical practice before their internship. Furthermore, the integration of virtual and physical teaching equipped the students with professional knowledge and evaluation skills related to AT for their future careers.

### Primary outcomes

The two groups did not differ significantly in their pretest scores, indicating that the groups had similar baseline knowledge. However, after the intervention, the experimental group exhibited a significant improvement of 20.67 points in the posttest, whereas the control group only improved by 3.67 points. This indicated that both groups improved their knowledge through the course but that the use of VR as a teaching tool resulted in significantly greater improvement. Although this finding is new in the field of AT, similar results have been obtained in other fields. One study [18] investigated medical students’ use of VR and attendance at interactive lectures to learn at university. The knowledge score obtained was significantly higher in the VR group than in the lecture group. Additionally, when VR was used in surgical skill training, students using VR exhibited superior learning efficiency and knowledge of orthopedic surgery [27]. Regarding nursing education, a systematic review [19] suggested that VR use can effectively improve student knowledge. These findings are consistent with our results.

A significant difference was discovered in the REA Form for Older Adults scores following the intervention in the experimental group, which scored 2.17 more points than did the control group, indicating that after the VR intervention, the students had superior skill learning outcomes than did those receiving the 2D video intervention. Although this result is new in the field of AT, similar results have been obtained in other fields. One study [31] investigated medical students’ use of VR and attendance of traditional lectures to learn skills related to infection control and identified that both methods improved the students’ skills but that the overall skill level achieved was higher in the VR group. This result suggests that VR is equally or more effective than traditional lectures for teaching skills related to infection control. Additionally, a study [17] comparing VR with lectures for medical students learning coma management skills found that the VR group obtained significantly superior learning outcomes to those of the lecture group, indicating that the VR intervention was effective in the medical students’ learning. When VR was used in surgical skill training, it was superior to technical video training for teaching complex procedural skills and critical steps in orthopedic surgery [27]. Generally speaking, when VR is employed to enhance skill levels, the VR group exhibits superior performance to the control group, although the result is not always significant [19, 29]. However, in our study, the skill level of the VR group significantly surpassed that of the 2D video group.

The course interventions in the aforementioned studies [17, 18, 31] differed substantially between the experimental and control groups, but in our course interventions, the same video was employed; the only difference was the immersion and interactivity provided by VR in the experimental group [5]. Therefore, our study more effectively demonstrates the learning benefits of VR immersion and interactivity.

### Secondary outcome

#### Positive feedback

1. Realism and Enjoyability: Students expressed that the VR experience was highly realistic, enjoyable, and easy to operate. This suggests that the immersive nature of the HMD-IVR positively impacted their engagement.
2. Enhanced Understanding: Participants noted that VR enabled them to more deeply understand the course content. This outcome indicates that the immersive technology contributed to a better comprehension of the educational material.
3. Empathy Building: VR was acknowledged for its ability to allow users to empathize with the challenges faced by older adults in daily life. This aspect highlights the potential of VR in fostering empathy and understanding in educational contexts.
4. Anticipation for Future Use: Overall, participants expressed anticipation for the future use of VR in various teaching and care scenarios. This outlook suggests a willingness to embrace VR as a valuable tool in educational settings.

#### Negative feedback

1. Cybersickness Symptoms: During HMD-IVR usage, three out of thirty students reported experiencing dizziness and nausea. The symptoms are mainly associated with cybersickness [48]; however, in this study, none of the students stopped using the device due to this issue and were able to complete the course. The incidence rate of these symptoms is relatively low compared to non-immersive VR.
2. Device Weight Complaints: Several students complained that the HMD-IVR device was too heavy. This feedback suggests a physical discomfort associated with the weight of the equipment, which could impact user experience.

### Recommendations for follow-up studies

First, we compared the difference in the effectiveness of IVR and traditional video teaching. Further research should explore whether the teaching effectiveness of IVR differs from that of non-immersive VR. Second, the participants in our study were third-year university students studying gerontology and long-term-care-related subjects. Future studies can enroll students in their freshman, sophomore, or senior year to explore whether the year of study affects future internship performance after the intervention. Third, we reproduced experiences of aging-related decline of sight and wheelchair use to simulate the conditions experienced by older adults and those with disabilities. In further research, more disabilities could be simulated, including mental and physical disabilities (i.e., using age or hemiplegia simulation suits).

### Study limitations

First, our study did not use random assignment to create groups; In addition, we did not assess whether the participants cohabited with individuals who used AT. Second, the intervention time for the VR group was longer than that for the video group. Third, there was no pretest and posttest comparison of the skills assessment. It could not be ensured that the abilities of the two groups were equal before the intervention. Fourth, only the VR group completed the Virtual Reality Experience Worksheet; it would have been necessary to design a 2D video experience worksheet to compare students’ perceptions of the interventions.

In this study, the students were required to attend class to experience immersive and interactive VR. Due to the outbreak of COVID-19, which has emphasized the increasing importance of distance learning, future experiments could explore the use of cardboard to enable students to use IVR at home [49]. While this approach may pose a challenge by potentially diminishing the level of interaction with the VR environment, its primary benefit lies in significantly enhancing the popularity and convenience of VR teaching. Effectively, it offers a valuable alternative during circumstances that require distance learning.

## Conclusion

VR experiential learning can significantly improve undergraduate students’ knowledge and evaluation skills in relation to AT for older adults and individuals with disabilities. The results of the present study can serve as a reference for gerontology and long-term-care-related education. VR experiential learning should be added to syllabi for undergraduate students to enhance their knowledge and evaluation skills and thus improve the quality of care and service they provide in future internships.

## Data Availability

The datasets used or analyzed during the current study are available from the corresponding author on reasonable request.

## Abbreviations

AC: Abstract conceptualization
AE: Active experimentation
AT: Assistive technology
CE: Concrete experience
HMD-IVR: Head-mounted display–immersive virtual reality
IVR: Immersive virtual reality
REA: Residential Environment Assessment
RO: Reflective observation
SPSS: Statistical package for social sciences
VR: virtual reality
